# Neurocinema: A brief overview

**Published:** 2015-07-06

**Authors:** Abdorreza Naser Moghadasi

**Affiliations:** ^1^MS Research Center, Neuroscience Institute, Sina Hospital, Tehran University of Medical Sciences, Tehran, Iran

**Keywords:** Neurocinema, Consciousness, Cognitive Science

## Abstract

Cinema is a multidimensional art capable of affecting our neurophysiologic structure in different ways. Studies show that different parts of the brain are activated while watching a structured film and consequently, the movie imitates consciousness structure. This imitation of the consciousness structure enables cinema to deeply influence the brain. The effect and its manner are the main themes of the newly-emerged science of neurocinema.

## Introduction

Since the advent of cinema, it has always been considered by scholars working on the mind and its functions. Perhaps the earliest points on the relationship between the cinema and the brain were mentioned by Henri Bergson, the great French philosopher. He exemplified cinema explaining what goes on in the mind in the book “Matter and Memory.” He tried to provide modern methods on thinking about movement through creating the concept of “movement-image.”^1^ In an intelligent theory proposed by Bertrand Russell, particularly when cinema was taking its first steps, he mentioned that cinema was the most important factor capable of destroying the free will. Russell truly referred to the effect of cinema on the mind and stated that many children learned about basic concepts of life like love, commitment, work, etc., through watching Hollywood movies and not by their free will and a thought unaffected by the elements of the modern world (such as cinema and modern education).^2^ Thinking about the very theory, it might be surprising that one could have such an intelligent understanding of the cinema about a century ago. Following the development of the cinema, directors have tried to make a further influence on the audience’s mind throughout this time. A clear example of the influence could be found in Hitchcock’s movies. His movies create a complicated psychological atmosphere and keep the audience suspended; therefore, he takes the opportunity to escape from the mind of the audience. “Vertigo” or “Rear Window” are good examples of the Hitchcock’s mentioned approach. Other great directors have their own methods of deeply influencing the mind of the audience. Would it be possible to watch Kurosawa’s Rashomon or Ran and not be influenced? This will be extensively discussed. There are several movies which show the interest of the directors and scriptwriters in neurologic diseases that can serve as a topic for a separate article. However, the present article is trying to find a deeper expression of the relationship between the brain and cinema. How can cinema affect the brain and where is the limit? Is Russell’s statement, a philosopher’s warning or something rooted in the extraordinary nature of the cinema?

**Cinema and Consciousness**

Prior to commenting on the cinema and its relationship with the brain, we should learn about this phenomenon. According to Jean-Luc Godard, cinema is a multidimensional art using different complicated stimuli such as music, sound, and picture.^3^ In addition to the mentioned stimuli, film editing has a significant role^4^ as it integrates numerous pictures and sounds to provide a consistent structure. The combination of these factors creates a complicated phenomenon that can affect the mind of the audience multi-dimensionally.

Perhaps one of the most considerable qualities of a movie is its flow. We are facing a series of events which are frequently and connectively targeting our minds like daily issues but with a purposeful and relevant structure. Is it possible to say that a filmmaking process copies and rebuilds the consciousness structure? In order to obtain a better understanding of the subject, consciousness, and its main qualities should be defined.

What is consciousness? Thomas Nagel answers this question in a short but very famous article entitled “What is it like to be a bat?”^5^ Nagel considers the subjective state of an experience. In order to clarify what he means, Nagel exemplified a bat (What is it like to be a bat?). As he further explains, bats have a sensory, cognitive system different from “humans.”

This sensory system based on reflection and echolocation enables the bat to recognize its surrounding world. It detects the distance, size, and form of an object in this way. Clearly, the system is quite different from the one that helps humans to know the external world. Nagel states that if this feeling is assumed as an experience by the bat, we should admit that there is something, and that is being a bat. In other words, Nagel holds that consciousness is tantamount to experience. What does the experience of seeing “red” look like? What does the experience of “smelling a flower” look like? This “look like” is determined by our consciousness. As Ned Block says, consciousness can be assumed a way through which we see, hear, or even experience the surrounding world.^6^ Therefore, the conscious experience can be considered as recognizing a thing.^7^ The consciousness plays an important role in our being as a human. Being exposed to a movie is a conscious experience because we are facing a multi-dimensional stimulator that can influence our cognitive abilities coherently and categorically. Indeed, the structural similarity of a movie to the concept of consciousness plays an important role.

In order to better understand the similarity, we need to study the formation of consciousness in humans from the point of neuroscience. According to the definition of William James, consciousness is a completely new concept. He refers to consciousness as a private, mental process that is continuous, purposeful, and unified^8^ and transcendental thought and awareness are also formed in such settings. The definition implies that consciousness is not induced by the outer world although the process of learning is an important pillar of the mind; however, it is finally “we” or “the conscious self” who learns and learning should take place inside, or in other words, in our brain. Consciousness is not something separate from us.

Accordingly, a new window is opened to the manner of developing consciousness through neuroscience.

Gerald Edelman proposed the most important theory of consciousness in neuroscience. He believes that consciousness does not refer to the activity of a certain area of the brain or a specific type of neurons; instead, it is the result of a dynamic, fluid relationship between a vast range of neurons. The thalamo-cortical system is a major structure for conscious activity. The system is not only internally connected but is also linked to other parts of the brain. On the other hand, the content of our consciousness is associated with different parts of the cortex. Neuroscientifically, this complex system forms our consciousness power. However, Edelman goes beyond the system in describing consciousness and is seeking a theory based on natural selection to explain how it is formed. He believes that the brain is the result of evolution, and the consciousness theory should also be based on the Darwinian evolution. Consequently, he put forward the theory of Theory of Neuronal Group Selection (TNGS) or the theory of neuronal group selection according to which numerous different cycles are formed as connections between neurons and different areas of the brain. Then, natural selection and Darwinism determine the neuronal cycle that would remain. The selection is done through synapses and their capabilities. Paths and links with more positive input that are applicable in human experiences and their communication with the surrounding world have a higher chance of survival.^9^ However, the gravity point of the TNGS theory as the generator of consciousness is a principle called the re-entry principle.^10^ Re-entry is a kind of exchange; a fluid exchange of information through a vast range of parallel axonal systems that connects maps and cores of the brain in a mirror-like manner.^10^ Consequently, a kind of synchronization is created among active cycles in all parts of the brain following the establishment of connections resulting from re-entry. Primary consciousness, like what is seen in more primitive organisms, turns into superior consciousness through a rich re-entrant activity between posterior and anterior parts of the brain with the ability to prioritize. This re-entrant activity is a neuronal basis for coordinating and creating what is called mental state or qualia.^10^

Edelman’s definition clarifies that consciousness involves different parts of the brain in one single experience. This involvement is not only an anatomic event but also the regions activate in a sequential order and fluid form; this is what happens during watching a movie. As cited from Jean-Luc Godard, a movie is a multi-dimensional process with different elements influencing it. Every scene is a series of pictures, sounds, music, and purposeful film editing; the scenes combine in a sequential order and create a fluid, unified ambiance, whose richness is a unique human experience, i.e., it associates a conscious experience with the mind. This is not just similarity but tests that confirm our hypothesis.

***Cinema on neuroscience tests***

The most important study conducted by Hasson et al. ^11^ examined the brain response and activity while watching a movie. He used fMRI in this study as well as a new method called “inter-subject correlation (ISC) analysis.” Using this method, the degree of similarity in brain activities of different viewers could be measured, which plays an important role in neurocinematic study because important aspects of studying brain responses to a movie are not equal to a neuroscientific response, and reactions of a considerable number of viewers need to be investigated. This can also be interesting in filmography and for professional film critics. Why are the opinions of critics about featured movies similar? Why are viewers sometimes fascinated by a movie? Why are some scenes memorable?

The study performed by Hasson et al.^11^ provides some answers to the questions. They showed that during watching movies like “The Good, the Bad, and the Ugly” as well as “Bang! You’re Dead,” ISC is remarkably higher when compared with scenes of everyday facts. This was also true in measuring the average eye movements of the viewers. The audience was free to look at any point while watching three different scenes. The study showed that eye fixation while watching the mentioned movies was considerably higher. The findings showed that a structured movie could significantly control the brain activity of the audience. In fact, ISC was high in a vast range of brain areas including the region related to vision, hearing, language perception (Wernicke’s area), feelings, and emotions as well as multi-sensory areas. This confirms the previous discussion on the cinema and statements of the great director, Jean-Luc Godard, who refers to cinema as a multidimensional art. It was actually quite predictable that this multidimensional art could influence different areas of the brain. This is the origin of the most important similarity between the cinema and consciousness structure.

***Role of cinema in transition and advancement of cognitive science***

Cognitive Science Movie Index (CSMI) is a valuable collection of famous movies that showcase various aspects of cognitive science.^12^ Motz properly pointed out that these movies could be tantamount to the cinematic examination of the mind.^12^ However, CSMI does not take into account the cultural factors affecting cinematic reception of the mind and the presence of cognitive science in movies is considered just due to its scientific points. Nevertheless, all cultures have the potential to change the manner of arguing in the mind according to their requirements. I intend to introduce a few most remarkable Iranian movies and evaluate the role of cinema in determining the effect of culture on cognitive structures of the mind.

In recent years, despite the growing development of the Iranian cinema,^13^ little attention has been paid to cognitive science. The Separation (2011) by Asghar Farhadi that won the Academy Award was one of the few Iranian movies that beautifully pictured Alzheimer’s disease and its impact on people’s lives. However, the impact of cognitive science on the Iranian cinema should be looked for in movies picturing internal conflicts of an Iranian individual that are undoubtedly an attribute of this cinema.

The identity of Iranian individuals has been influenced by the conflict between tradition and modernity. An Iranian person tries to find a relationship between a past loaded with historic honors, mysticism, and poem and the present world that is based on modernity and rationality; this conflict may sometimes create great conflicts in his mental and cognitive structures. Therefore, movies including similar issues would include the most important concepts of cognitive science for describing the mind of this individual. Ballad of Tara (1979) and Half Moon (2006) are simpler examples that show the contrast between two different worlds of historic past and mysticism and realities of the present world, respectively. This contrast practically brings about confusion for the (movie) hero. In a more thoughtful movie named Maybe Some Other Time (1987), a woman is after her past in her mind, and the search is full of nightmares and vague footprints in her memory. The movie demonstrates how nightmares and indistinct memories could change cognitive structures of the woman’s mind. Yet, the most distinguished movie with this theme might be Hamoon (1990). Hamoon demonstrates the life of an Iranian intellectual depicting his internal conflicts and portraiting his fluctuations among faith and unbelief, love and hatred, and tradition and modernity. As he cannot make a rational relationship between them, the nightmares do not abandon him, his power of reasoning is impaired, and all his cognitive structures collapse.

Although the Iranian cinema does not purely peruse scientific issues, it can be considered a kind of a cognitive test showing how cultural issues can change cognitive structures of the mind, which reminds us of the theories of Levy-Bruhl^14^ and Luria.^15^

CSMI can provide valuable opportunities for those interested in cognitive science to study the impact of culture on cognitive structures of the mind and also offer a way for an inter-cultural conversation.

***Future of neurocinema***

**Figure 1:**
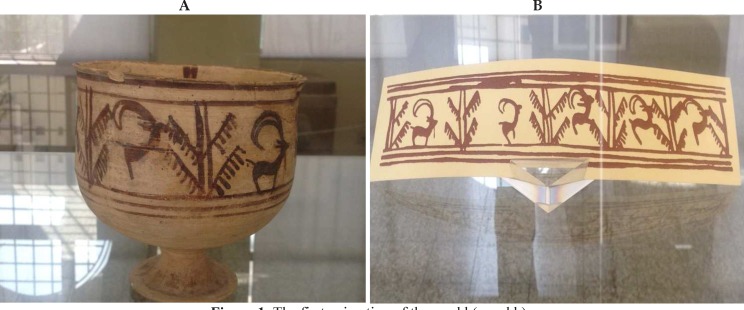
The first animation of the world (a and b)

It is now quite evident that neurocinema is opening a fascinating window in front of us. Perhaps the most important aspect of this study is to show the influence of cinema on the brain and its imitation of the structures of consciousness. This point has roots in the history and may even go further back to the prehistoric era. Studying the oldest animation of the world, currently preserved in the National Museum of Iran, explains this claim. The animation properly describes the present status of the cinema (Figures 1 a and b). A goat on a bowl approaches a tree in a few episodes and finally eats the leaves.^16^ Before this, movement or movement-image, as mentioned at the beginning of the Bergson’s article, was not a concern in human productions. A single image like what is seen in historic caves can stimulate different brain areas but can by no means remind the concept of movement in mind while what is seen on the Burnt City Bowl stimulates different parts of the brain at different stages and creates a kind of sequential order in the mind. This order, i.e., having one image or situation coming over another, reminds us of the concept of movement in the mind. It should be noted that this movement and, according to what was mentioned earlier, the continuous communication between one situation and another are the main principles of consciousness. The Burnt City’s bowl could be considered as the first attempts of humans to control the mind, or in other words, the consciousness structure of the audience. Accordingly and in a historical approach, cinema is the effort of humans to manipulate and manage human consciousness, an effort that is continuously and seriously continuing. It seems that the application of new techniques has not only evolved the structure of the cinema but has also changed its effect on our mind and consciousness. Perhaps now, we can better understand the statements attributed to Bertrand Russell. What Russell refers to as the surprising effect of the cinema on our mind is not merely a moral concern but has roots in physiological bases of our brain and what is called neurocinema.

## Conclusion

Neurocinema is a newly emerged field examining the relationship between the cinema and the brain. On the first look, the most superficial layer of the relationship includes several movies with the theme of various brain diseases; however, a deeper look suggests the manner in which the brain is influenced by the cinema on the one hand and the response of the brain to other movies on the other hand. Neuroscience is a powerful tool for studying phenomenon which can influence our mind and cinema is one of them. Neurocinema as a multidisciplinary science can help us to study the relationship between movies and minds. In addition, neurocinema provides a new field to create opportunities for planning tests to confirm or reject different hypotheses on cultural differences and brain functions in different cultures.

## Notes:

**How to cite this article: **Naser Moghadasi A. Neurocinema: A brief overview. Iran J Neurol 2015; 14(3): 180-4.
